# Supplementary material for: Do birds of a feather leave the nest together? The role of sibling personality similarity in the transition to adulthood
Source: PLoS One. 2023 May 17;18(5):e0284808. doi: 10.1371/journal.pone.0284808 (PMC10191368; doi:10.1371/journal.pone.0284808)
Supplement: S1 File — (DOCX) [file pone.0284808.s001.docx]

**Supporting information**

**Supplementary analysis 1**

The full table of Table 2 in the main text can be found in Table S1.

| Table S1. Unstandardized coefficients of the multilevel discrete-time event-history analysis predicting at-risk children’s event of leaving (*N* time level = 33,612) | | | | | | |
| --- | --- | --- | --- | --- | --- | --- |
|  | Model 1 | | Model 2 | | Model 3 | |
|  | b | se | b | se | b | se |
| Intercept | -21.980 | 1.317*** | -21.532 | 1.338*** | -24.059 | 2.047*** |
| Age | 1.423 | 0.109*** | 1.422 | 0.109*** | 1.416 | 0.109*** |
| Age^2^ | -0.023 | 0.002*** | -0.023 | 0.002*** | -0.023 | 0.002*** |
| ***Family/parental level characteristics*** | | | | |  |  |
| Education (ref: low) |  |  |  |  |  |  |
| High | 0.624 | 0.132*** | 0.618 | 0.131*** | 0.621 | 0.132*** |
| Middle | 0.301 | 0.112** | 0.292 | 0.111** | 0.297 | 0.111** |
| Unknown | -0.240 | 0.237 | -0.219 | 0.236 | -0.206 | 0.236 |
| Employment (ref: no) |  |  |  |  |  |  |
| Yes | 0.045 | 0.084 | 0.055 | 0.084 | 0.051 | 0.084 |
| Unknown | -0.912 | 0.228*** | -0.906 | 0.228*** | -0.889 | 0.228*** |
| Relationship status  (ref: divorced/separated) | |  |  |  |  |  |
| Married/cohabiting | -0.181 | 0.081* | -0.179 | 0.081* | -0.180 | 0.080* |
| Unknown | -0.576 | 0.210** | -0.580 | 0.210** | -0.594 | 0.210** |
| Number of children | 0.010 | 0.038 | 0.016 | 0.038 | 0.011 | 0.038 |
| ***Child level characteristics*** | | | | |  |  |
| Gender (ref: male) | 0.648 | 0.091*** | 0.645 | 0.090*** | 0.643 | 0.091*** |
| Education (ref: low) |  |  |  |  |  |  |
| High | -0.204 | 0.159 | -0.207 | 0.159 | -0.205 | 0.159 |
| Middle | -0.296 | 0.142* | -0.301 | 0.141* | -0.300 | 0.141* |
| Unknown | -0.394 | 0.278 | -0.399 | 0.277 | -0.394 | 0.277 |
| Employment (ref: no) |  |  |  |  |  |  |
| Yes | 0.152 | 0.069* | 0.153 | 0.069* | 0.149 | 0.069* |
| Unknown | -0.420 | 0.247 | -0.419 | 0.247 | -0.428 | 0.246 |
| Relationship status (ref: single) |  |  |  |  |  |  |
| In a relationship | 0.718 | 0.225** | 0.721 | 0.225** | 0.734 | 0.226** |
| Unknown | 0.627 | 0.118*** | 0.631 | 0.118*** | 0.628 | 0.118*** |
| Child’s Big Five traits |  |  |  |  |  |  |
| Extraversion | 0.060 | 0.040 | 0.061 | 0.040 | 0.445 | 0.171** |
| Conscientiousness | 0.034 | 0.043 | 0.046 | 0.043 | 0.155 | 0.199 |
| Agreeableness | -0.055 | 0.045 | -0.064 | 0.046 | -0.116 | 0.217 |
| Openness | -0.116 | 0.038** | -0.120 | 0.038** | -0.268 | 0.130* |
| Neuroticism | 0.054 | 0.034 | 0.051 | 0.034 | 0.208 | 0.094* |
| ***Sibling dyad level characteristics*** | | | | |  |  |
| Biological sibling (ref: no) | -0.563 | 0.186** | -0.542 | 0.185** | -0.573 | 0.185** |
| Siblings’ age difference | -0.042 | 0.013** | -0.042 | 0.013** | -0.043 | 0.013** |
| Sibling left (ref: no) | 0.741 | 0.063*** | 0.025 | 0.569 | 3.597 | 2.256 |
| Sibling’s Big Five traits |  |  |  |  |  |  |
| Extraversion | -0.007 | 0.031 |  |  | 0.404 | 0.169* |
| Conscientiousness | -0.034 | 0.035 |  |  | 0.039 | 0.198 |
| Agreeableness | 0.010 | 0.036 |  |  | -0.041 | 0.216 |
| Openness | -0.046 | 0.029 |  |  | -0.197 | 0.127 |
| Neuroticism | -0.014 | 0.025 |  |  | 0.111 | 0.096 |
| Sibling similarity in the Big Five |  |  |  |  |  |  |
| Similarity in extraversion |  |  | -0.094 | 0.047* |  |  |
| Similarity in conscientiousness |  |  | -0.094 | 0.052 |  |  |
| Similarity in agreeableness |  |  | 0.017 | 0.053 |  |  |
| Similarity in openness |  |  | 0.036 | 0.045 |  |  |
| Similarity in neuroticism |  |  | -0.046 | 0.040 |  |  |
| ***2-way Interactions*** |  |  |  |  |  |  |
| Sibling similarity in the Big Five and sibling left |  |  |  |  |  |  |
| Similarity in E x Sibling left |  |  | 0.144 | 0.064* |  |  |
| Similarity in C x Sibling left |  |  | 0.004 | 0.069 |  |  |
| Similarity in A x Sibling left |  |  | 0.061 | 0.070 |  |  |
| Similarity in O x Sibling left |  |  | -0.103 | 0.060 |  |  |
| Similarity in N x Sibling left |  |  | 0.041 | 0.053 |  |  |
| Child’s Big Five and sibling’s Big Five |  |  |  |  |  |  |
| Child’s E x Sibling’s E |  |  |  |  | -0.073 | 0.035* |
| Child’s C x Sibling’s C |  |  |  |  | -0.014 | 0.038 |
| Child’s A x Sibling’s A |  |  |  |  | 0.011 | 0.039 |
| Child’s O x Sibling’s O |  |  |  |  | 0.025 | 0.026 |
| Child’s N x Sibling’s N |  |  |  |  | -0.030 | 0.023 |
| Child’s Big Five and sibling left |  |  |  |  |  |  |
| Child’s E x Sibling left |  |  |  |  | -0.582 | 0.227* |
| Child’s C x Sibling left |  |  |  |  | -0.025 | 0.264 |
| Child’s A x Sibling left |  |  |  |  | -0.038 | 0.302 |
| Child’s O x Sibling left |  |  |  |  | 0.258 | 0.174 |
| Child’s N x Sibling left |  |  |  |  | -0.140 | 0.129 |
| Sibling’s Big Five and sibling left |  |  |  |  |  |  |
| Sibling’s E x Sibling left |  |  |  |  | -0.620 | 0.227** |
| Sibling’s C x Sibling left |  |  |  |  | 0.092 | 0.259 |
| Sibling’s A x Sibling left |  |  |  |  | -0.066 | 0.304 |
| Sibling’s O x Sibling left |  |  |  |  | 0.242 | 0.174 |
| Sibling’s N x Sibling left |  |  |  |  | -0.057 | 0.127 |
| ***3-way interactions*** |  |  |  |  |  |  |
| Child’s Big Five, sibling’s Big Five, sibling left |  |  |  |  |  |  |
| Child’s E x Sibling’s E x Sibling left |  |  |  |  | 0.102 | 0.046* |
| Child’s C x Sibling’s C x Sibling left |  |  |  |  | -0.019 | 0.050 |
| Child’s A x Sibling’s A x Sibling left |  |  |  |  | 0.011 | 0.054 |
| Child’s O x Sibling’s O x Sibling left |  |  |  |  | -0.037 | 0.036 |
| Child’s N x Sibling’s N x Sibling left |  |  |  |  | 0.011 | 0.032 |
| Unexplained variances family level | 0.798 | 0.149*** | 0.777 | 0.148*** | 0.759 | 0.146*** |
| Unexplained variances child level | 2.980 | 0.222*** | 2.953 | 0.221*** | 2.957 | 0.222*** |
| * p< .05 ** p< .01 *** p< .001. | | | | |  |  |

**Supplementary analysis 2**

As a first robustness check, we randomly selected one sibling dyad per child using simple random sampling and eliminated the sibling dyad level. With the remaining three levels (the family, child, and time/wave level), we repeated the multilevel discrete-time event-history analysis for Model 2 and 3 in Table 2. As shown in Table S2, the results with the three-level data structure greatly resembled the main findings.

| Table S2. Unstandardized coefficients of the multilevel discrete-time event-history analysis with one randomly selected sibling dyad per child (*N* time level = 24,898) | | | | |
| --- | --- | --- | --- | --- |
|  | Model 2 | | Model 3 | |
|  | b | se | b | se |
| Intercept | -12.216 | 1.327*** | -16.653 | 2.111*** |
| ***Child level characteristics*** | | |  |  |
| Child’s extraversion | 0.041 | 0.033 | 0.620 | 0.171*** |
| ***Sibling dyad level characteristics*** | | |  |  |
| Sibling left (ref: no) | -0.692 | 0.604 | 6.481 | 2.373** |
| Sibling’s extraversion |  |  | 0.561 | 0.172** |
| Sibling similarity in extraversion | -0.124 | 0.047** |  |  |
| ***2-way Interactions*** |  |  |  |  |
| Sibling similarity in extraversion x Sibling left | 0.172 | 0.066** |  |  |
| Child’s extraversion x Sibling’s extraversion |  |  | -0.110 | 0.035** |
| Child’s extraversion x Sibling left |  |  | -0.746 | 0.235** |
| Sibling’s extraversion x Sibling left |  |  | -0.734 | 0.235** |
| ***3-way interactions*** |  |  |  |  |
| Child’s extraversion x Sibling’s extraversion x Sibling left |  |  | 0.127 | 0.048** |
| Unexplained variances family level | 0.245 | 0.113* | 0.235 | 0.112* |
| Unexplained variances child level | 1.857 | 0.172*** | 1.856 | 0.172*** |
| *Note:* All covariates in Table S1 were included as well. For simplicity and clarity, they were not shown in the table. * p< .05 ** p< .01 *** p< .001. | | | | |

**Supplementary analysis 3**

We conducted a sensitivity analysis using fixed-effects (FE) modeling by using the family identification as a covariate, creating (n – 1) dummies for each household (1). Furthermore, given that repeated measures may be observed for at-risk children with more than one sibling, a random intercept at the child level was included in the models as well. While time-invariant variables at the family level were removed from the models, as they were accounted for by the family-level fixed effects, time-varying family level characteristics were kept in the model. With this design, we again repeated Model 2 and 3 in Table 2. Like the first additional analysis, this additional check provided us with results corresponding to the main analysis (see Table S3). Even if the effect of a sibling who had left became negative, if a nest-leaving sibling was observed, the at-risk children were still more likely to leave if they had a similar level of extraversion, especially for two introverts.

| Table S3. Unstandardized coefficients of the discrete-time event-history analysis with fixed effects at the family level and random effects at the child level (*N* time level = 33,612) | | | | |
| --- | --- | --- | --- | --- |
|  | Model 2 | | Model 3 | |
|  | b | se | b | se |
| Intercept | -27.472 | 2.044*** | -31.425 | 3.129*** |
| ***Child level characteristics*** | | |  |  |
| Child’s extraversion | 0.043 | 0.049 | 0.550 | 0.235* |
| ***Sibling dyad level characteristics*** | | |  |  |
| Sibling left (ref: no) | -1.709 | 0.585** | 2.112 | 2.424 |
| Sibling’s extraversion |  |  | 0.512 | 0.230* |
| Sibling similarity in extraversion | -0.190 | 0.065** |  |  |
| ***2-way Interactions*** |  |  |  |  |
| Sibling similarity in extraversion x Sibling left | 0.206 | 0.065** |  |  |
| Child’s extraversion x Sibling’s extraversion |  |  | -0.102 | 0.047* |
| Child’s extraversion x Sibling left |  |  | -0.756 | 0.246** |
| Sibling’s extraversion x Sibling left |  |  | -0.815 | 0.245*** |
| ***3-way interactions*** |  |  |  |  |
| Child’s extraversion x Sibling’s extraversion x Sibling left |  |  | 0.150 | 0.050** |
| Unexplained variances child level | 1.155 | 0.164*** | 1.912 | 0.292*** |
| *Note:* Family level fixed effects using (n – 1) dummies for each family (estimates not shown) were included in the model. All covariates in Table S1 were included as well (except for time-invariant family level characteristics). For simplicity and clarity, they were not shown in the table. * p< .05 ** p< .01 *** p< .001. | | | | |

**Supplementary analysis 4**

To further test whether the interaction effects of extraversion were confounded by agreeableness, a proxy of relationship quality, we stratified our sample based on siblings’ agreeableness and repeated Model 2 and 3 in Table 2. When both the at-risk children and their siblings scored 5 or above on agreeableness, they were counted as agreeable sibling dyads. When they scored below 5, they represented the unagreeable dyads.

| Table S4. Unstandardized coefficients of the multilevel discrete-time event-history analysis stratified by siblings’ agreeableness | | | | | | | | |
| --- | --- | --- | --- | --- | --- | --- | --- | --- |
|  | Model 2 | | Model 3 | | Model 2 | | Model 3 | |
|  | Agreeable siblings | | | | Unagreeable siblings | | | |
|  | b | se | b | se | b | se | b | se |
| Intercept | -17.551 | 1.636*** | -23.012 | 2.524*** | -19.364 | 1.825*** | -19.782 | 2.402*** |
| ***Child level characteristics*** | | |  |  |  |  |  |  |
| Child’s extraversion | 0.070 | 0.047 | 0.806 | 0.235*** | 0.016 | 0.050 | 0.042 | 0.218 |
| ***Dyad level characteristics*** | | |  |  |  |  |  |  |
| Sibling left (ref: no) | 0.051 | 0.677 | 8.127 | 2.775** | 0.459 | 0.719 | 0.138 | 2.313 |
| Sibling’s E |  |  | 0.803 | 0.234*** |  |  | 0.003 | 0.216 |
| Sibling similarity in E | -0.071 | 0.061 |  |  | -0.104 | 0.063 |  |  |
| ***2-way Interactions*** |  |  |  |  |  |  |  |  |
| Sibling similarity in E x Sibling left | 0.155 | 0.084**^+^** |  |  | 0.138 | 0.087 |  |  |
| Child’s E x Sibling’s E |  |  | -0.144 | 0.047** |  |  | 0.001 | 0.046 |
| Child’s E x Sibling left |  |  | -1.196 | 0.320*** |  |  | 0.017 | 0.296 |
| Sibling’s E x Sibling left |  |  | -1.246 | 0.321*** |  |  | -0.025 | 0.297 |
| ***3-way interactions*** |  |  |  |  |  |  |  |  |
| Child’s E x Sibling’s E x Sibling left |  |  | 0.226 | 0.064*** |  |  | -0.021 | 0.062 |
| Unexplained variances family level | 0.452 | 0.152** | 0.447 | 0.153** | 0.477 | 0.163** | 0.462 | 0.160** |
| Unexplained variances child level | 2.125 | 0.233*** | 2.132 | 0.235*** | 1.973 | 0.255*** | 1.946 | 0.254*** |
| *N* time level | 19,379 | | | | 14,233 | | | |
| *Note:* All covariates in Table S1 were included as well, except for the variables and terms related to agreeableness. For simplicity and clarity, they were not shown in the table. **^+^** p<.01 * p< .05 ** p< .01 *** p< .001. | | | | | | | | |

**Supplementary analysis 5**

After accounting for parental agreeableness (proxy for parent-child relationship quality) and official regions in the UK in which the family resided (i.e., the parental home), the effects found in Table 2 were still present (see Table S5 below).

| Table S5. Unstandardized coefficients of the multilevel discrete-time event-history analysis including parental agreeableness and UK regions (*N* time level = 27,061) | | | | |
| --- | --- | --- | --- | --- |
|  | Model 2 | | Model 3 | |
|  | b | se | b | se |
| Intercept | -22.444 | 1.455*** | -25.099 | 2.221*** |
| ***Family/parental level characteristics*** |  |  |  |  |
| Parental agreeableness | -0.021 | 0.052 | -0.016 | 0.052 |
| Region (ref: Northern Ireland) |  |  |  |  |
| North East | 0.574 | 0.296 | 0.567 | 0.295 |
| North West | 0.859 | 0.211*** | 0.870 | 0.210*** |
| Yorkshire and the Humber | 0.626 | 0.225** | 0.617 | 0.225* |
| East Midlands | 1.214 | 0.236*** | 1.200 | 0.235*** |
| West Midlands | 0.239 | 0.222 | 0.235 | 0.222 |
| East of England | 0.519 | 0.223* | 0.529 | 0.222* |
| London | -0.634 | 0.228** | -0.648 | 0.228** |
| South East | 0.610 | 0.197** | 0.624 | 0.197** |
| South West | 0.710 | 0.231** | 0.700 | 0.230** |
| Wales | 0.728 | 0.201** | 0.708 | 0.200** |
| Scotland | 0.634 | 0.208** | 0.627 | 0.208** |
| ***Child level characteristics*** | | |  |  |
| Child’s extraversion | 0.030 | 0.042 | 0.400 | 0.183* |
| ***Sibling dyad level characteristics*** | | |  |  |
| Sibling left (ref: no) | -0.733 | 0.600 | 4.331 | 2.355 |
| Sibling’s extraversion |  |  | 0.391 | 0.180* |
| Sibling similarity in extraversion | -0.094 | 0.051 |  |  |
| ***2-way Interactions*** |  |  |  |  |
| Sibling similarity in extraversion x Sibling left | 0.163 | 0.067* |  |  |
| Child’s extraversion x Sibling’s extraversion |  |  | -0.023 | 0.041 |
| Child’s extraversion x Sibling left |  |  | -0.602 | 0.237* |
| Sibling’s extraversion x Sibling left |  |  | -0.638 | 0.238** |
| ***3-way interactions*** |  |  |  |  |
| Child’s extraversion x Sibling’s extraversion x Sibling left |  |  | 0.110 | 0.048* |
| Unexplained variances family level | 0.817 | 0.155*** | 0.794 | 0.153*** |
| Unexplained variances child level | 2.816 | 0.233*** | 2.810 | 0.233*** |
| *Note:* All covariates in Table S1 were included as well. For simplicity and clarity, they were not shown in the table. * p< .05 ** p< .01 *** p< .001. | | | | |

**Supplementary analysis 6**

In this additional analysis, we tested the non-linear effects of all Big Five traits, by adding the quadratic terms. We found that only the quadratic term of at-risk children’s conscientiousness made an additional contribution (see Fig. S1): if a sibling with low conscientiousness had left, an at-risk child was more likely to leave if he/she was conscientious. Also, if a sibling who was highly conscientious had left, an at-risk child scoring low in conscientiousness was more prone to leaving. However, this increased flexibility of an already complex model also heightens the risk of overfitting, thus especially this finding requires replication before firm conclusions can be made.


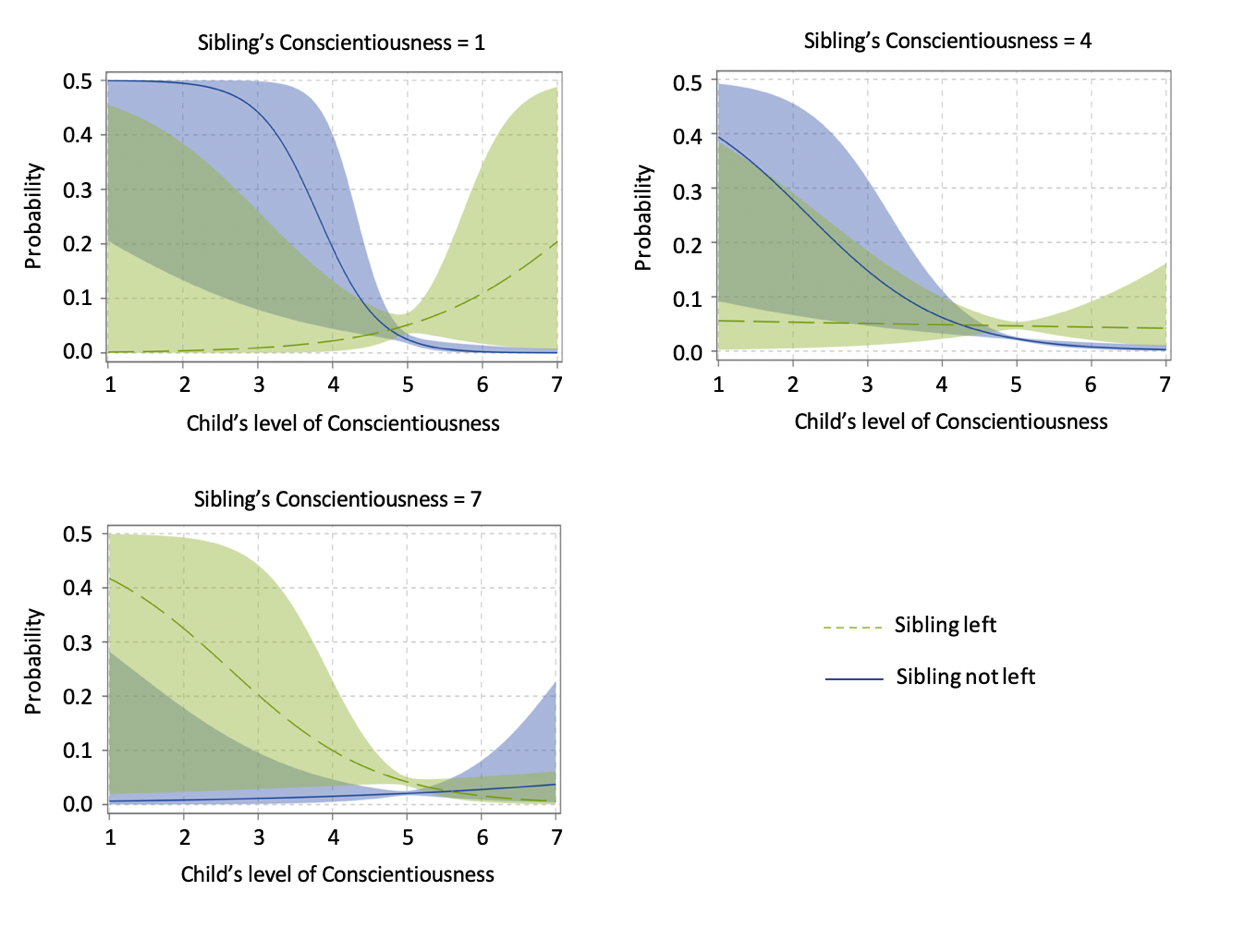


**Fig S1. The association between siblings’ departures as moderated by child’s conscientiousness and sibling’s conscientiousness.** The quadratic terms associated with conscientiousness were modeled as well. (Predicted probabilities for event = 1, with 95% confidence limits).

**References**

1. Allison PD. Fixed effects regression models: SAGE publications; 2009.
