# Supplementary material for: Do birds of a feather leave the nest together? The role of sibling personality similarity in the transition to adulthood
Source: PLoS One. 2023 May 17;18(5):e0284808. doi: 10.1371/journal.pone.0284808 (PMC10191368; doi:10.1371/journal.pone.0284808)
Supplement: Attachment — Submitted filename: Review PONE-D-22-27514.pdf [file pone.0284808.s002.pdf]

This article studies how sibling similarity in their personality is associated to sibling nest leaving. The authors theorize on sibling influence, the behavior of one sibling affecting the other, and they use ‘paving the way’ arguments, hypothesizing that siblings who are more similar in their personality are more likely to move out once the first sibling has moved out. The authors conclude that similarity in extraversion, especially when both are introverts, was associated to stronger associations between one’s own nest leaving and that of the sibling.

Main points

The authors clearly explain the importance of timely nest leaving for adolescents, but why is it important to study *dyadic* nest leaving? Why would siblings with similar personalities leave together? I understand that influence plays a role, but given the dyadic approach in this paper, I would have expected some more reasoning as to why sibling nest leaving is not a matter of age or simply the sum of the decision of two individuals, or a result of the personality of two individuals. Are these decisions truly that dependent? I think the paper could be improved in the introduction/background and discussion section by stressing the importance and contribution of studying dyadic nest leaving more.

Before one could argue that similar siblings “leave the nest together” it seems important to control for the quality of the sibling relationship as well as the quality of the intergenerational relationship, i.e., siblings liking each other may postpone moving out (together) or when they do not like each other enjoy time home alone with their parents. Siblings not being close to their parents may be encouraged to leave the nest early regardless of their (similar) personalities. Lastly, could you control for the distance to the first sibling moving out? Effects may differ when parents live in a rural area and the first sibling moves out to a large university city or when parents were already living in such a city and the first sibling moves out moves to a student flat nearby.

It was not clear to me whether this data set is a multi-actor dataset. Are the personality traits etc. reported by both siblings? If the data are multi-actor, are the reports of both siblings first considered as ‘child’ and next as ‘sibling’? Related to this is the question how should I understand the child at risk? Does this mean that only children are considered of which at least one sibling has already left the household? In that case the reports of both siblings cannot be used once as child and once as sibling. While some effort has certainly already been made, I would like some more explanation of the data structure and implications for analysis.

The authors argue that similarity on extraversion especially holds for both siblings being introverts (page 19-20). From which numbers do the authors derive this conclusion? Moreover, from what score on extraversion are you considered to be an introvert? Perhaps robustness checks could be performed dichotomizing this variable on the introvert cut-off score to see whether the results hold.

Smaller points

- The abstract starts with a reference to social network studies, but this is not quite the focus of the article. Therefore, the second sentence does not logically follow from this first sentence.
- In the background section some references are made to homophily and social network studies, but some major references to social network studies about homophily are lacking, such as

McPherson, M., Smith-Lovin, L., & Cook, J. M. (2001). Birds of a feather: Homophily in social networks. *Annual Review of Sociology*, *27*, 415–444. https://doi.org/10.1146/annurev.soc.27.1.415

- “Siblings were identified based on mother’s lineage” (page 10). What does that mean?
- The structure of the sample description (page 9-11) could be improved. My suggestion would be to first discuss the inclusion criteria, then all decisions made and to end with the N of the analytical sample.
- “… those who had not left the nest at the age of 29 were right censored” (page 11). How many observations did you lose with this decision?
- Related to this at page 11: “53.8% of the children at risk had an event”. Does this mean that 46% still lives at home? What age are they?
- Page 13: “… we selected the mother. Otherwise, we took the available biological parent as the targeted parent”. How often does this occur? Are there any implications of this decision?
- Why are the main effects of sibling personality not reported in model 2?
- Instead of reporting the full table with all two-way and three-way interactions in the main text, the authors could consider to report a slimmed version of the table and to report the extended version in an online Appendix. I am no expert on these analyses, but would it for example be an option to report linear combinations of coefficients instead of the interactions?
- Sometimes you refer to odds (e.g., page 20 “the odds of leaving decreased”), whereas b’s are reported in table 2. Is this correct? Could you show the SE’s in addition to the significance stars?
- Figure 1: this figure is not mentioned in the text. Is the description of level 1 correct? It refers to the dyad level at level 3, but shouldn’t this be level 2?
