# Supplementary material for: Do birds of a feather leave the nest together? The role of sibling personality similarity in the transition to adulthood
Source: PLoS One. 2023 May 17;18(5):e0284808. doi: 10.1371/journal.pone.0284808 (PMC10191368; doi:10.1371/journal.pone.0284808)
Supplement: Attachment — Submitted filename: Review PONE-D-22-27514-R1.pdf [file pone.0284808.s004.pdf]

PONE-D-22-27514-R1

I wish to thank the authors for their effort in improving the manuscript and their thorough reply letter. Below I reply to the authors' reply to my comments in case I wish to see any further clarifications. Meaning that if I did not address a comment, I have no further remarks and I am satisfied with the changes made.

Overall, I found the manuscript improved. I did notice quite some spelling errors though, especially in the newly written text. I would encourage the authors to thoroughly re-read the paper another time and/or to consider a language check. The mistakes I spotted are listed below.

Comment 1 (importance of the study):
The authors elaborated on the arguments for nest leaving similarity in the background section. They also stressed the benefits of doing this with a dyadic approach in the methods section. But I am still missing a statement (at the end of) the introduction why it is important to study dyadic nest leaving. What puzzle that we were not able to solve can now – perhaps due to your dyadic approach – be solved? Please add this statement.

Comment 2 (quality of sibling and intergenerational relationships):
"After accounting for parental agreeableness (proxy for parent-child relationship quality) and official regions in the UK in which the family resided, the effects found in Table 2 were still present (see Table A5 below)." Does family here refer to the residence of the parents and the siblings that are still living at home or does it also include the region of the sibling who moved out? From the appendices it seems that only the residence of the parental home is considered, so this does not tell us something about distance between siblings and distance between siblings and parents yet. Please clarify.

Comments 3 (multiple sibling reports per family):
"At-risk children's siblings may or may not be living in the household when they had an event." Although this paragraph is clearer, this sentence was not clear to me. Why are children who are experiencing the event of moving out not necessarily living in the parental home? Also, what does "they" refer to? Please adjust the sentence.

Comment 5 (abstract)
"Empirical evidences on intragenerational transmission of life course have been demonstrated and that interpersonal similarity may moderate the effect." Although the abstract has improved by changing this sentence, this sentence is not really clear.

My suggestion would be to write: *"Empirical studies on the intragenerational transmission of life course events have demonstrated that …. Our hypothesis is that sibling similarity moderates this effect."*

Comment 9 and 10 9 (those not experiencing the event)
Great you added the numbers. It would be great if you could also add one sentence whether and how it affects the results that not all children experienced the event yet.

Spell checks:

The running title of the manuscript, why is not “similarity in **the** transition to adulthood”?

Abstract
…, particularly when they were both introvert**s. (or scored high on introversion)**

… which helps explain**ing** young adults’ home-leaving decision in an
era when delayed leaving is observed.

Introduction
… and whether one has a nest-leaving sibling also play**s** a role in shaping this transition (7, 8).

Theoretical background

The focus of our study was **[is?]** to examine which factors moderate intragenerational transmission of leaving, specifically how personality similarity functions as a potential determinant.

Data & methods
The mean age of children **in** was 19.77 (*SD* = 3.19) in wave 15 and 20.56 (*SD* = 3.33) in wave 21.

Other double checks

- Is it right that the majority of parents was divorced, separated or single? (Table 1)
- Unexplained variance at the child level > 1 in Table 2, is that correct?
- Rather than ‘confirming hypotheses’ consider ‘supporting hypotheses’
